# Supplementary material for: Disparities in COVID-19 hospitalizations and mortality among black and Hispanic patients: cross-sectional analysis from the greater Houston metropolitan area
Source: BMC Public Health. 2021 Jul 6;21:1330. doi: 10.1186/s12889-021-11431-2 (PMC8258471; doi:10.1186/s12889-021-11431-2)
Supplement: Supplementary file 1 — Additional file 1: Supplemental Table 1. Socio-Demographic, Comorbidity, and Clinical Factors Associated with In-Hospital Mortality among Hospitalized COVID-19 Patients. Supplemental Table 2. Baseline Socio-Demographic and Comorbidity Factors Associated with Hospitalization among SARS-CoV-2 Positive Individuals. Supplemental Table 3. Differences in COVID-19 Case Demographics for Select U.S. Metropolitan Areas. [file 12889_2021_11431_MOESM1_ESM.docx]

**Disparities in COVID-19 Hospitalizations and Mortality among Black and Hispanic Patients: Cross-Sectional Analysis from the Greater Houston Metropolitan Area**

Alan P. Pan MS,^1^ Osman Khan BS,^1^ Jennifer R. Meeks MS,^1^

Marc L. Boom MD,^2,3^ Faisal N. Masud MD,^3,4^ Julia D. Andrieni MD,^2,3^

Robert A. Phillips MD PhD,^1,3,5^ Yordanos M. Tiruneh MPhil PhD,^6^

Bita A. Kash PhD MBA,^1,3,7^ Farhaan S. Vahidy PhD MBBS MPH^1,8^

*Center for Outcomes Research, Houston Methodist, Houston TX*

*Department of Clinical Medicine, Houston Methodist, Houston TX*

*Weill Cornell Medical College, New York NY*

*Department of Anesthesiology and Critical Care, Houston Methodist, Houston TX*

*Department of Cardiology, Houston Methodist, Houston TX*

*University of Texas Health Science Center at Tyler, TX*

*Texas A&M University School of Public Health, College Station, TX*

1. *Houston Methodist Neurological institute, Houston Methodist, Houston, TX*

**Corresponding Author**

Farhaan S. Vahidy, PhD MBBS MPH FAHA

Associate Professor and Associate Director

Center for Outcomes Research, Houston Methodist

Josie Roberts Administration Building

7550 Greenbriar Drive, Suite 4.123

Houston, TX 77030

Phone: 346.356.1479 | Email: fvahidy@houstonmethodist.org

ORCID: 0000-0002-3464-2111

**Supplemental** **Table 1**. Socio-Demographic, Comorbidity, and Clinical Factors Associated with In-Hospital Mortality Among hospitalized COVID-19 patients

**Supplemental** **Table 2**. Baseline Socio-Demographic and Comorbidity Factors associated with hospitalization among SARS-CoV-2 Positive Individuals

**Supplemental** **Table 3**. Differences in COVID-19 Case Demographics for Select U.S. Metropolitan Areas

**Supplemental Table 1:** Socio-Demographic, Comorbidity, and Clinical Factors Associated with In-Hospital Mortality Among hospitalized COVID-19 patients

|  | **Mortality**  *(n = 234)* | **No Mortality**  *(n = 3,302)* | **OR (95% CI)^a^** |
| --- | --- | --- | --- |
| **Demographic and Social Characteristics – n (%)** | | | |
| Age – mean (SD) | 73.4 (13.4) | 58.0 (17.1) | 1.06 (1.05, 1.07) |
| Female (vs. Male) | 103 (44.0) | 1592 (48.2) | 0.84 (0.65, 1.10) |
| Race |  |  |  |
| White | 143 (61.1) | 2,007 (60.8) | *Reference* |
| Black | 58 (24.8) | 889 (26.9) | 0.92 (0.66, 1.25) |
| Asian | 18 (7.7) | 128 (3.9) | 1.97 (1.14, 3.25) |
| Other / Mixed / Not Reported | 15 (6.4) | 278 (8.4) | 0.76 (0.42, 1.27) |
| Hispanic (vs. non-Hispanic) | 66 (28.2) | 1,300 (39.4) | 0.60 (0.45, 0.81) |
| Insurance Type |  |  |  |
| Commercial | 25 (10.7) | 1,159 (35.1) | *Reference* |
| Medicare | 103 (44.0) | 1,244 (37.7) | 3.84 (2.50, 6.11) |
| Medicaid | 1 (0.4) | 207 (6.3) | 0.22 (0.01, 1.07) |
| Self-Pay | 10 (4.3) | 623 (18.9) | 0.74 (0.34, 1.51) |
| Other | 95 (40.6) | 69 (2.1) | 63.83 (39.18, 107.48) |
| Residence: Low Income ZIP Code^b^ | 120 (51.3) | 1,824 (55.2) | 0.84 (0.64, 1.10) |
| Residence: High Pop Density ZIP Code^c^ | 98 (41.9) | 1,501 (45.5) | 0.86 (0.65, 1.12) |
| **Comorbidities and Pre-existing Conditions – n (%)** | | | |
| Charlson Comorbidity Index Score | 7 (5 – 9) | 3 (1 – 6) | 1.21 (1.18, 1.25) |
| BMI – mean (SD) | 29.6 (7.5) | 31.5 (8.2) | 0.97 (0.95, 0.99) |
| Obesity | 83 (35.5) | 1,031 (31.2) | 1.21 (0.91, 1.59) |
| Hypertension | 205 (87.6) | 2,136 (64.7) | 3.86 (2.64, 5.84) |
| Smoking (Current/Former) | 82 (35.0) | 862 (24.0) | 1.84 (1.38, 2.45) |
| **Vital Signs at Hospital Admission – n (%)** | | | |
| SBP (mmHg) – mean (SD) | 133.5 (22.5) | 133.3 (19.3) | 1.00 (0.99, 1.01) |
| DBP (mmHg) – mean (SD) | 69.1 (10.3) | 72.7 (9.6) | 0.96 (0.95, 0.97) |
| Respiratory Rate ≥ 24 breath / min | 76 (32.5) | 665 (20.1) | 1.91 (1.43, 2.53) |
| Temperature ≥ 38°C | 10 (4.3) | 217 (6.6) | 0.52 (0.23, 0.99) |
| Oxygen Saturation < 94% | 69 (29.5) | 545 (16.5) | 2.14 (1.58, 2.86) |
| **Hospital Complications – n (%)** | | | |
| Pneumonia | 192 (82.1) | 2168 (65.7) | 2.39 (1.72, 3.41) |
| ARDS | 66 (28.2) | 123 (3.7) | 10.15 (7.23, 14.19) |
| Bronchitis | 6 (2.6) | 38 (1.2) | 2.26 (0.85, 5.02) |
| Lower Respiratory Tract Infection | 0 (0.0) | 33 (1.0) | - |
| Acute Renal Injury | 153 (65.4) | 625 (18.9) | 8.09 (6.11, 10.78) |
| Acute Hepatic Injury | 23 (9.8) | 23 (0.7) | 15.54 (8.55, 28.27) |
| Cardiomyopathy or CHF | 82 (35.0) | 340 (10.3) | 4.70 (3.50, 6.27) |
| Hypoxic Respiratory Failure | 133 (56.8) | 1250 (37.9) | 2.16 (1.66, 2.83) |
| **Therapeutics – n (%)** | | | |
| Hydroxychloroquine | 58 (24.8) | 305 (9.2) | 3.24 (2.34, 4.43) |
| Ribavirin | 35 (15.0) | 69 (2.0) | 8.24 (5.31, 12.60) |
| Azithromycin | 21 (9.0) | 508 (15.4) | 0.54 (0.33, 0.84) |
| Lopinavir/Ritonavir | 9 (3.8) | 15 (0.5) | 8.77 (3.65, 19.92) |
| Remdesivir | 25 (10.7) | 666 (20.2) | 0.47 (0.30, 0.71) |
| Tocilizumab | 75 (32.1) | 608 (18.4) | 2.09 (1.56, 2.78) |
| Antithrombotic | 115 (49.1) | 1,058 (32.0) | 2.05 (1.57, 2.68) |
| Anticoagulants | 209 (89.3) | 2,981 (90.3) | 0.90 (0.60, 1.42) |
| Dexamethasone | 79 (33.8) | 1,473 (44.6) | 0.63 (0.48, 0.83) |
| **Laboratory Parameters – n (%)^d^** | | | |
| WBC count <4000/μl | 5/234 (2.1) | 230/3293 (7.0) | 0.29 (0.10, 0.64) |
| Lymphocytes < 20% | 214/234 (91.5) | 2147/3291 (65.2) | 5.70 (3.68, 9.35) |
| Platelet count <150,000/μl | 65/234 (27.8) | 370/3293 (11.2) | 3.04 (2.23, 4.11) |
| B-natriuretic peptide >100 pg/ml, | 144/220 (65.5) | 680/2275 (29.9) | 4.44 (3.33, 5.98) |
| Procalcitonin >0.25 ng/ml | 109/135 (80.7) | 521/1137 (45.8) | 4.96 (3.23, 7.87) |
| Troponin ≥ 0.06 ng/ml | 164/221 (74.2) | 500/1798 (27.8) | 1.03 (1.01, 1.05) |
| Aspartate aminotransferase > 40 U/l | 174/232 (75.0) | 1625/3227 (50.4) | 2.95 (2.19, 4.04) |
| Alanine aminotransferase >40 U/l | 107/232 (46.1) | 1352/3221 (42.0) | 1.18 (0.90, 1.55) |
| Total Bilirubin ≥ 1.2 mg/dl | 26/223 (11.7) | 104/3009 (3.5) | 3.69 (2.30, 5.72) |
| C-reactive protein >8.2 ng/ml | 206/207 (99.5) | 2663/2812 (94.7) | 11.53 (2.57, 203.25) |
| Ferritin level > 3000 ng/ml | 33/206 (16.0) | 148/2845 (5.2) | 3.48 (2.28, 5.17) |
| D-dimer > 0.5 ug/ml | 197/198 (99.5) | 2342/2779 (84.3) | 36.76 (8.25, 646.93) |
| Creatinine > 1.5 mg/dl | 115/234 (49.1) | 487/3264 (14.9) | 5.51 (4.19, 7.25) |
| Venous lactate > 2.2 mmol/l | 103/217 (47.5) | 397/2351 (16.9) | 4.45 (3.33, 5.93) |
| **Hospital Acuity of Care Factors – n (%)** | | | |
| ICU admission | 190 (81.2) | 911 (27.6) | 11.33 (8.17, 16.05) |
| Invasive Mechanical Ventilation | 160 (68.4) | 421 (12.7) | 14.80 (11.07, 19.94) |
| *^a^ Unadjusted Odds Ratios and 95% Confidence Intervals for association between individual co-variates and in-hospital mortality among confirmed COVID-19 cases*  *^b^ Low income classified as ZIP codes in the 2 lowest pentiles for median house income*  *^c^ High population density classified as ZIP codes in the 2 highest pentiles for population density*  *^d^ Denominator denotes number of non-missing values*  *SBP: Systolic Blood Pressure, DBP: Diastolic Blood Pressure, ARDS: Acute Respiratory Distress Syndrome, CHF: Congestive Heart Failure, WBC: White Blood Cell Count* | | | |

**Supplemental Table 2:** Baseline Socio-Demographic and Comorbidity Factors associated with hospitalization among SARS-CoV-2 Positive Individuals

|  | **Hospitalized**  *(n = 3,536)* | **Not Hospitalized**  *(n = 8,548)* | **OR (95% CI)^a^** |
| --- | --- | --- | --- |
| **Demographic and Social Characteristics – n (%)** | | | |
| Age – mean (SD) | 59.0 (17.3) | 41.7 (16.7) | 1.06 (1.06, 1.06) |
| Female (vs. Male) | 1,695 (47.9) | 5033 (58.9) | 0.64 (0.59, 0.70) |
| Race |  |  |  |
| White | 2,150 (60.8) | 4918 (57.5) | *Reference* |
| Black | 947 (26.8) | 2150 (25.2) | 1.01 (0.92, 1.10) |
| Asian | 146 (4.1) | 464 (5.4) | 0.72 (0.59, 0.87) |
| Other / Mixed / Not Reported | 293 (8.3) | 1016 (11.9) | 0.66 (0.57, 0.76) |
| Hispanic (vs. Non-Hispanic) | 1,366 (38.6) | 3006 (35.2) | 1.12 (1.04, 1.22) |
| Insurance Type |  |  |  |
| Commercial | 1,184 (33.5) | 4615 (54.0) | *Reference* |
| Medicare | 1,347 (38.1) | 864 (10.1) | 6.08 (5.46, 6.76) |
| Medicaid | 208 (5.9) | 534 (6.2) | 1.52 (1.28, 1.80) |
| Self-Pay | 633 (17.9) | 2433 (28.5) | 1.01 (0.91, 1.13) |
| Other | 164 (4.6) | 102 (1.2) | 6.27 (4.86, 8.11) |
| Residence: Low Income ZIP Code^b^ | 1,944 (55.0) | 3940 (46.1) | 1.44 (1.33, 1.56) |
| Residence: Population Dense ZIP^c^ | 1,599 (45.2) | 3776 (44.2) | 1.05 (0.97, 1.14) |
|  | | | |
| Charlson Comorbidity Index Score | 3 (1 – 6) | 0 (0 – 2) | 1.40 (1.37, 1.42) |
| BMI – mean (SD) | 31.4 (8.1) | 30.8 (7.3) | 1.01 (1.00, 1.01) |
| Obesity | 1,114 (31.5) | 1326 (15.5) | 2.50 (2.28, 2.74) |
| Hypertension | 2,341 (66.2) | 2280 (26.7) | 5.38 (4.95, 5.86) |
| Smoking (Current/Former) | 874 (24.7) | 1263 (14.8) | 1.49 (1.35, 1.65) |
| *^a^ Unadjusted Odds Ratios and 95% Confidence Intervals for association between individual co-variates and hospitalization among confirmed COVID-19 cases*  *^b^ Low income classified as ZIP codes in the 2 lowest pentiles for median house income*  *^c^ High population density classified as ZIP codes in the 2 highest pentiles for population density* | | | |

**Supplemental Table 3.** Differences in COVID-19 Publicly Reported Case Demographics for Select U.S. Metropolitan Areas

|  | **Houston** | **New Orleans** | **New York City** | **Los Angeles** | **Chicago** | **United States** |
| --- | --- | --- | --- | --- | --- | --- |
| **Total Cases / Hospitalizations / Deaths**^a^ | 94,978 / - / 1,491  (~7/28/2020) | 10,071 / - / 553  (~7/28/2020) | 220,907 / 56,040 / 18,878  (~7/28/2020) | 178,642 / ~12,300 / 4,426  (~7/28/2020) | 59,388 / - / 2,759  (~7/27/2020) | 4.2M / 147K  (~7/28/2020) |
| **Infection** | | | | | | |
| Ratio (NHB% / NHW%) | 0.84  (22.0 / 26.1) | 3.20  (60.2 / 18.8) | 1.05  (29.9 / 28.5) | 0.37  (4.2 / 11.4) | 1.94  (29.9 / 15.4) | - |
| Ratio (Hispanic% / NH%) | 0.85  (45.9 / 54.1) | - | 0.52  (34.0 / 66.0) | 1.26  (55.7 / 44.3) | 0.88  (46.9 / 53.1) | - |
| **Hospitalization** | | | | | | |
| Ratio (NHB% / NHW%) | - | - | 1.33  (33.0 / 24.9) | - | - | - |
| Ratio (Hispanic% / NH%) | - | - | 0.52  (34.4 / 65.6) | - | - | - |
| **Mortality** | | | | | | |
| Ratio (NHB% / NHW%) | - | 3.25  (75.1 / 23.1) | 1.09  (30.4 / 27.8) | 0.42  (10.6 / 25.2) | 2.24  (43.0 / 19.2) | - |
| Ratio (Hispanic% / NH%) | - | - | 0.51  (33.6 / 66.4) | 0.92  (47.8 / 52.2) | 0.49  (32.7 / 67.3) | - |
| **Population Size and Demographics** | | | | | | |
| Population, 2018^b^ | 2,325,502 | 391,006 | 8,398,748 | 3,990,456 | 2,705,994 | 328,239,523 |
| Region Demographics^b^ | | | | | | |
| White only, not Hispanic/Latino | 24.6% | 30.6% | 32.1% | 28.5% | 32.8% | 60.4% |
| Black/AA only | 22.5% | 59.7% | 24.3% | 8.9% | 30.1% | 13.4% |
| Black / White Ratio | 0.91 | 1.95 | 0.76 | 0.31 | 0.92 | 0.22 |
| Hispanic/Latino | 44.8% | 5.5% | 29.1% | 48.6% | 29.0% | 18.3% |
| Hispanic / Non-Hispanic Ratio | 0.81 | 0.06 | 0.41 | 0.95 | 0.36 | 0.22 |
| *^a^* *Source:*  *https://www.cdc.gov/coronavirus/2019-nCoV/index.html*  *https://www.tmc.edu/coronavirus-updates/*  *https://publichealth.harriscountytx.gov/Resources/2019-Novel-Coronavirus* *https://ready.nola.gov/incident/coronavirus/*  *https://www1.nyc.gov/site/doh/covid/covid-19-data.page*  *http://publichealth.lacounty.gov/media/Coronavirus/*  *https://www.chicago.gov/city/en/sites/covid-19/home.html*  *Note: Public data are reported. Information not available is left blank.*  *^b^* *American Community Survey, 2014-18* | | | | | | |
